# Use of Beach Shoes for Foot Protection during the Bangkok Flood of 2011

**DOI:** 10.5704/MOJ.1303.007

**Published:** 2013-03

**Authors:** Saranatra Waikakul

**Affiliations:** Department of Orthopaedic Surgery, Faculty of Medicine Siriraj Hospital, Bangkok, Thailand

## Abstract

*Background:* Foot injury was common as a result of the Bangkok flood of 2011. In the future, this type of injury should be prevented to lessen the burden during a disaster. *Objectives:* The study was performed to ascertain what type of footwear is appropriate for volunteer rescue workers during a flood. *Material and Methods:* The study was carried out during the flood in November 2011 at Siriraj Hospital. There were 15 volunteers enrolled in the study. None of the volunteers had any foot deformity or injury before the study. Participants were divided into 3 groups of 5 volunteers: group A, the barefoot group; group B, the high top shoe group; and group C, the beach shoe group. All volunteers worked in the areas close to Siriraj Hospital and were followed up after 5 days of rescue work. Prevalence of foot and ankle injuries, satisfaction regarding work conditions and willingness to use the shoes were subjectively evaluated. Wearing of beach shoes during rescue was satisfactory during the early phase of the flood. *Results:* The age range of volunteers was 20-28. In the group A, most volunteers were barely satisfied with conducting rescue work in water with bare feet, that bare feet were good for working on a wet surface and were ‘just satisfied’ to not satisfied that bare feet were good for work on dry surfaces. In group B, most of the volunteers had opinions similar to group A with the exception that they felt better while they were working on dry surfaces. In group C, most volunteers were significantly more satisfied under all three conditions. Foot injury occurred in 2 volunteers from group A. *Conclusion:* Beach shoes offer adequate foot protection during flood rescue.

## Introduction

During the catastrophic flood in Bangkok in 2011, foot and ankle injuries were among the more common musculoskeletal injuries seen in emergency clinics. Lacerations, puncture wounds and ankle sprain were the most common foot injuries.

In the early days of the flood, when the water level was rising rapidly and strong currents were present, most flood victims were evacuated quite rapidly from their homes. These people and the volunteers who were involved in the disaster have to move as fast in floodwaters as possible to save lives and property. Most walked or ran in the water on their bare feet as they felt that they could move more freely with no shoes. Further, it is common practice for the people in Thailand to walk and run on bare feet during work in the water such as farming and fishing; however, doing so during a natural flood disaster may render volunteer rescue workers prone to injury^1^.

Some workers used high top shoes, but the weight of the shoes slowed movement when walking in water. Furthermore, the height of high top shoes usually was not sufficient as the water level increased very rapidly during the early phase of the flood, meaning that water overflowed into the shoes, making walking quite difficult. Some workers used athletic shoes because they could walk or run in the water more smoothly than with high top shoes. However, the athletic shoes also became wet and the cushion of the shoes usually separated from the inner part of the shoes after wearing in the water for a short period of time. Furthermore, athletic shoes were slippery when walking on a wet surface or in the water, and possibly resulted in injuries to wearers of such shoes.

**Fig. 1:**
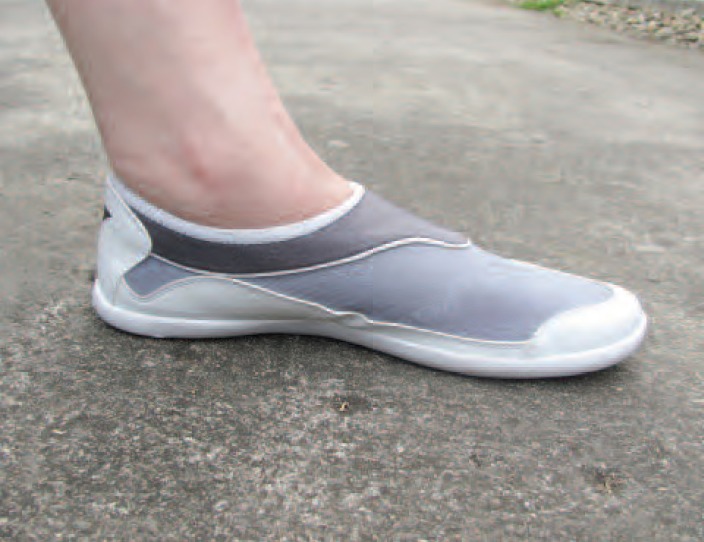
: The beach shoes securely fit to the feet and are made of strong and durable rubber.

**Fig. 2:**
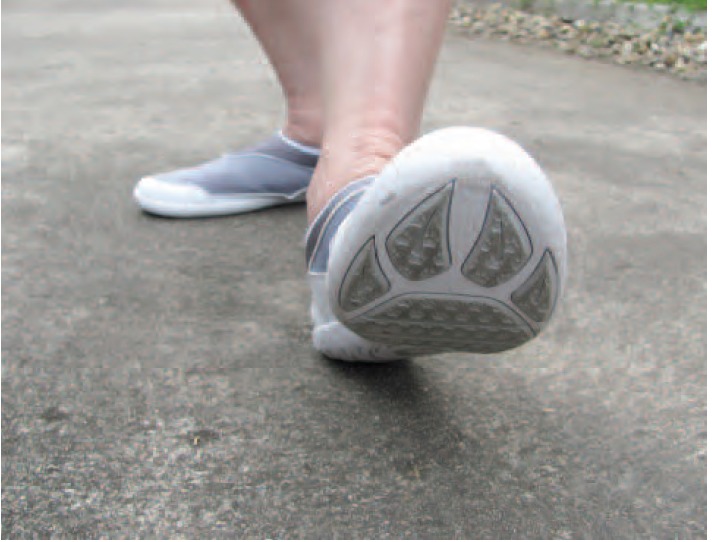
: The shoes have grip the floor well.

Beach shoes or water shoes are designed for walking on the beach and in water. The shoes are made of strong and durable rubber to protect feet from sharp objects such as spiny shells, horse mussels and broken sea shells, (Figure 1). The shoes also have good floor-grip to prevent slipping during walking or climbing on submerged rocks (Figure 2). The aim of this study was to compare the usefulness of beach shoes in the protection of foot and ankle injuries during working in the flood. Satisfaction of individuals who wore this type of beach shoe for while walking or running in the flood was also evaluated subjectively.

## Materials and Methods

The study was a control trial with a 5 day follow up as the Bangkok flood came and went quite rapidly and the trial was carried out during that period of urgency. The study sample consisted of 15 volunteers who worked as flood rescuer in the area around Siriraj Hospital and Aroon Ummarind Junction (about 500 meters north of the hospital) in November 2011. All volunteers were male and 12 of the 15 were government employees. None had any foot deformity or injury before participating in the trial. To accommodate participation, the volunteers divided into groups by their own selection (3 groups of 5 volunteers per group; see Table I). In group A all volunteers conducted rescue work in bare feet, while in group B all volunteers used high top shoes with the upper rim at their knees or above. In group C all volunteers used beach shoes which were supplied by study staff (Figure 1). All were followed every 1 to 2 days and underwent final evaluation at the end of the 5th working day. Participants were questioned about foot and ankle injuries that occurred while working in the floodwaters. The investigators examined feet and ankles as well. Volunteers were queried as to their level of satisfaction with the shoes they wore or with working in bare feet; they were asked about subjective satisfaction when working in water, on wet surfaces and on dry surfaces. We also asked volunteers if they would continue to use shoes (or bare feet) as they did during the study. There were 5 possible replies to questions about level of satisfaction: very good, good, just satisfied, not satisfied and poor. After the final evaluation, the volunteers exchange views about their experiences working in bare feet, water shoes or high top shoes. Prevalence of foot and ankle injuries during the study was also recorded.

Data was analysed using the student t-test for continuous data and analysis of variance was used to analyse discrete numbers.

## Results

All volunteers completed the study. There was no significant difference among the volunteers in terms of their age (Table I). All volunteers worked between 8 to 10 hours a day. In group A, most of the volunteers responded that they were barely satisfied using bare feet while working in water, good while working on wet surfaces and just satisfied to not satisfied while they were working on dry surfaces. Group B volunteers responded similarly to group A in terms of working in the water and on wet surfaces, but felt better than group A while they were working on dry surfaces. Most group C volunteers reported significantly higher levels of satisfaction under all rescue conditions. Most group A and group B volunteers said they would like to change to beach shoes if they were to continue to work as a volunteers. None in group C reported that they would like to use bare feet or high top shoes in further work (Table I).

**Fig. 3:**
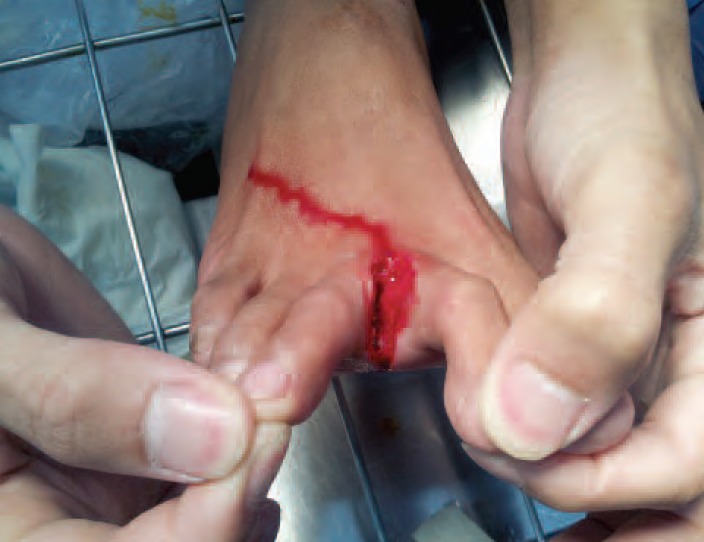
: One volunteer from group A experienced a laceration at the second web of the right foot.

Foot injury was found in 2 volunteers from group A. One had a small laceration on the plantar surface of his left foot and the other one had a laceration at the second web of his right foot, (Figure 3). Two volunteers in group B experienced water over flow into the high top shoes during the work period (Table I).

## Discussion

Limitations of this study include the limited number of study participants, the short observation timeframe and that most data was subjective. Since the flood in Bangkok happened unexpectedly, this study was carried out with some urgency. This led to the necessarily small group sizes and the short term for follow-up. Most volunteers had to move to other affected flood zones to continue to rescue those people affected by floods. Furthermore, all volunteers were very busy during the flood period and did not spend enough time participating in the subjective evaluation sessions. Further, as subjects self selected their study group, there was probably selection bias that affected the results.

Volunteers in groups A and B reported lower satisfaction than the volunteers in the group C, especially during the early phases of the flood when water levels increased rapidly and the current was quite strong. Although volunteers in group A felt that bare feet could provided freedom for walking and running in water, they worried about potential foot injuries. Most windows and doors of buildings in the flood area were broken by the current, meaning there was a high risk of foot wounds in the disaster workers^2^. Two participants in group A experienced foot lacerations (Figure 3), and all group A volunteers reported slippery surfaces even though none experienced a major fall and no foot or ankle sprains were observed. Furthermore, group A participants working on dry surfaces complained of pain and discomfort.

Volunteers in group B complained about the weight and resistance of the high top shoes for working in water. Due to quickly changing water levels, the height of the high top shoes did not always protect their feet from moisture. Two volunteers in group B experienced water overflow into their high top shoes due to work in deep water areas.

On the other hand, volunteers in group C reported higher levels of satisfaction working in all conditions compared to groups A and B. All group C volunteers felt that the beach shoes offered good foot protection for all three conditions, namely in water, on wet surfaces and on dry surfaces. However, beach shoes do not prevent leg, knee or thigh injuries from flood debris. High top shoes might provide better protection than the beach shoes from such debris. During steady and late stages of flood, the beach shoe might not be suitable as it does not prevent skin contact with contaminated and dirty water, which in turn may result in dermatitis and skin infection. During the steady stage and recovery from flood, high top shoes might be the foot protectors of choice.

Compared to bare feet, beach shoes have similar resistance regarding movement in water. Volunteers felt that the beach shoes gripped securely in the 3 environments. None experienced slipping and falling down during work. Those in group C reported that they could walk and run with comfort in the water. Furthermore, they felt that cleaning of the beach shoes was quite simple. None in the group C reported that they would change to high top shoes or bare feet in a future rescue operations.

Beach shoes have rather high prices (ranged, 800-1,200 baht) whereas the range of price for high top shoes is wider (200- 2,000 baht), depending on the shoe materials. For instance, plastic high top shoes prices were between 100 and 300 baht, but did not provide a good grip when working in water. Prices for high top shoes increased up 3 to 5 times during the flood, probably due to high demand.

**Table I:**
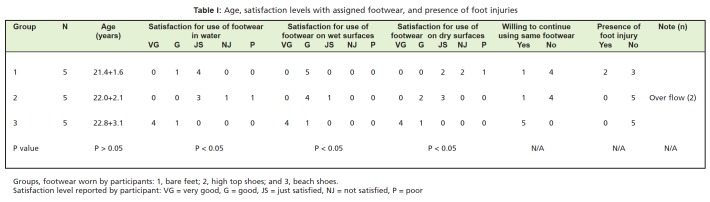
: Age, satisfaction levels with assigned footwear, and presence of foot injuries

## Summary

During early phases of the flood, the beach shoe seemed to be the most suitable footwear; however, a larger study sample and long follow up periods should be used to confirm our results.
